# Machine learning-based study of the predictors of clinically important change in patient-reported outcomes in bilateral upper-limb function in patients receiving robotic stroke rehabilitation

**DOI:** 10.3389/fresc.2026.1738402

**Published:** 2026-01-26

**Authors:** Yu-Wen Chen, Keh-chung Lin

**Affiliations:** 1 Department of Speech-Language Pathology and Audiology, National Taipei University of Nursing and Health Sciences, Taipei, Taiwan; 2 School of Occupational Therapy, College of Medicine, National Taiwan University, Taipei, Taiwan; 3 Department of Physical Medicine and Rehabilitation, Division of Occupational Therapy, National Taiwan University Hospital, Taipei, Taiwan; 4 Department of Occupational Therapy, College of Medical Science and Technology, Chung Shan Medical University, Taichung, Taiwan

**Keywords:** activities of daily living, machine learning, patient-reported outcome measures, prognosis, robotics, stroke rehabilitation, upper extremity

## Abstract

**Introduction:**

Robotic therapy is an effective approach for poststroke rehabilitation of upper-limb function. There is a need for research on predictors of clinically meaningful change in patient-reported outcome measures of bilateral arm function relevant for daily life function in stroke patients receiving robotic therapy.

**Methods:**

This secondary analysis included data from 123 participants who received robotic therapy. We constructed machine learning classification models to predict the achievement of meaningful recovery of patient-perceived outcome of bilateral upper limb function based on the ABILHAND questionnaire. Clinically meaningful recovery was defined using the minimal clinically important difference (MCID). The prediction models included 14 potential predictors of three categories: demographic characteristics, stroke characteristics, and baseline assessment scores. The prediction models were built using four algorithms: logistic regression, k-nearest neighbors, support vector machine, and the random forest. Full models were built with all 14 potential predictors, and parsimonious models were built with the most important predictors identified by feature selection.

**Results:**

The prediction accuracy of the best-performing full models was 0.76 for the anchor-based MCID and 0.80 for the distribution-based MCID; the corresponding areas under the receiver operating characteristic curve were 0.75 and 0.84, respectively. The parsimonious models performed worse overall. For both MCID values, the most important predictors of recovery were time since stroke, the Wolf Motor Function Test-Time score, and the Stroke Impact Scale-Physical function score. When recovery was defined by the anchor-based MCID, the baseline ABILHAND and Chedoke Arm and Hand Activity Inventory scores were among the most important predictors. When recovery was defined by the distribution-based MCID, sex and stroke diagnosis were predictive of recovery.

**Conclusions:**

The time post onset of stroke, the speed of performing functional tasks, and self-perceived physical function were identified as the most important predictors of improvements in self-perceived function of bilateral upper limbs in daily activities after robotic therapy. The findings may inform clinicians about characteristics of patients with stroke who are more likely to benefit from robotic therapy.

## 1 Introduction

Stroke is a leading cause of disability (1) that requires continued rehabilitation seeking recovery. Robotic therapy is an effective approach for upper-limb motor function rehabilitation (2). A recent systematic review (2) found improvements in motor impairments measured by the Fugl-Meyer assessment in patients receiving rehabilitative interventions, although this improvement did not achieve the minimal clinically important difference. Despite the popularity of rehabilitation robotics, there is insufficient evidence regarding the ability of robotic therapy to improve activities of daily living in stroke. Stroke characteristics may be associated with rehabilitation outcomes and warrant study. This study investigated the characteristics of people who are more likely to recover after post-stroke robotic therapy.

Studies on prognosis prediction usually involve pretreatment assessments and other characteristics of participants to predict treatment outcomes. Both clinician-rated scales and patient-reported outcome measures (PROMs) are used in assessments of recovery status. Clinician-rated scales focus on expert perspectives of body movement and function. Clinicians are trained to evaluate movement and function in a standardized way. Studies on prognosis prediction frequently focus on these clinician-rated outcomes. However, as the recent focus of healthcare practice has shifted toward patient-centered care, rehabilitation programs have increasingly focused on activities of daily living. This aspect of rehabilitation cannot be precisely measured via clinician-rated scales. The nature of clinician-rated and patient-reported scales entails that differences exist when these scales are used to define recovery, and different factors may contribute to the achievement of recovery according to each type of scale (3, 4). Thus, there is a need to further understand the factors that predict patient outcomes based on PROMs.

Unlike clinician-rated scales, PROMs are based on how people with stroke rate their own movement and function within the context of a personal history and their own daily activities and environment. PROMs are also closely related to an individual's views of their disability, culture, and personality. While clinician-rated scales provide a common language for interprofessional communication, PROMs offer valuable insights into the perspectives of people with stroke. Understanding the perspectives of people with stroke can promote their continuous participation in rehabilitation and contribute to the design and implementation of patient-centered rehabilitation programs. One frequently used patient-rated scale for quantifying functional recovery in stroke rehabilitation is the ABILHAND (5). The ABILHAND is a 46-item patient-reported scale that assesses patient-perceived stroke recovery with a focus on bilateral arm function in daily life activities. All the items assess tasks for which bilateral arm activity is critical. The ABILHAND is interview-based, where the person with a stroke is asked to estimate their ease or difficulty performing a given task involving bilateral arm activity without human or technical help. The ABILHAND is frequently used in clinical trials to quantify patient-perceived recovery after robotic therapy (6, 7) and has been shown to have a moderate-to-high correlation with patient-reported use of the affected arm in daily activities as rated by the Motor Activity Log (8).

Machine learning-based study of outcome prediction may inform precision rehabilitation. Machine learning enables the development of prediction models within clinics, and these models can be used to predict rehabilitation outcomes for a new person with a stroke. The factors that most strongly influence the accuracy of prediction models can be identified during model construction, and prediction scores obtained for a given new person with a stroke could be used to aid in clinical decision making and tailor the needs of the person with a stroke in the development of an individualized rehabilitation program (9). Given that PROMs are often associated with a wide range of dynamic and multidimensional factors or predictors (3), machine learning approaches may offer advantages in terms of predicting the prognosis or treatment outcomes defined by these measures.

Data analysis using machine learning approaches is data driven. This analysis involves the use of algorithms to find patterns in the input dataset and the construction of models to predict the variables of interest. The variable being predicted is called the target, and the potential predictors in the dataset are called features. One of the most common machine learning tasks is classification, where the target is a categorical variable. In classification, the goal of the models is to find patterns in the features to predict the category of individual data points. Compared with traditional statistical methods, machine learning approaches can take large amounts of data at once and conduct multidimensional and nonlinear data analyses. Additionally, machine learning approaches do not require extensive *a priori* knowledge about the features (10).

Previous studies on stroke rehabilitation have demonstrated the feasibility of using machine learning to predict prognoses and postintervention outcomes (11–19). These studies investigated a variety of outcome variables across the three domains of the International Classification of Functioning, Disability and Health (ICF) of the World Health Organization (20). Given the increasing emphasis on patient-centered care, we conducted a study using machine learning techniques to predict four PROMs of activities and participation following stroke rehabilitation and to identify important predictors of these PROMs (9). We reported the predictors of postintervention improvements in the Stroke Impact Scale (SIS), Motor Activity Log, and Nottingham Extended Activities of Daily Living scores. Previous studies have supported the feasibility of machine learning approaches for predicting prognosis in stroke rehabilitation. It was demonstrated that machine learning approaches are effective in analyzing dynamic and multifaceted PROMs. Additionally, we found that parsimonious models, i.e., models built with a subset of features, can perform equally well as models that include a larger number of features.

In accord with Chen et al. (9), the analyzed outcome measures of activities and participation encompassed a broader range of activities of daily living and did not exclusively focus on upper-limb function, which is the target of our rehabilitation programs. Furthermore, Chen et al. (9) analyzed data from studies on the effects of multiple therapy programs in stroke. It remains unclear which factors might predict patient-perceived outcome in bilateral upper limb function following robotic stroke rehabilitation. To address the research gap, this study was designed to 1) identify the most important predictors for clinically important change in the ABILHAND scores after robotic therapy and 2) construct machine learning classification models for the prediction of treatment outcome.

Prior research revealed that baseline assessment scores on a given PROM are among the most important predictors of postintervention improvements in this measure, which is consistent with earlier reports (11–13, 15, 21). Baseline upper limb motor function, particularly scores from the upper extremity subscale of the Fugl-Meyer assessment (FMA-UE), is also an important predictor of PROMs of activities and participation, which aligns with findings from previous reports (22, 23). Previous studies have also reported that these scores are important predictors of motor function (21, 24, 25). Demographic characteristics (e.g., age) and stroke characteristics (e.g., severity and time since stroke) have also been found to be important predictors of postintervention outcomes. Therefore, we included these relevant variables as potential predictors in the current study.

## 2 Material and methods

### 2.1 Study design, participants, intervention, and assessment

In this study, we analyzed data collected in our previous (6) and recent (NCT04446273) studies. The inclusion criteria were: 1) at least 3 months after the onset of a first-ever unilateral cerebral stroke; 2) a baseline FMA-UE score >10; 3) the ability to follow instructions; 4) a modified Ashworth scale spasticity score ≤3, indicating mild to moderate spasticity; and 5) no other orthopedic or neurologic disorders that impact upper-limb mobility. The exclusion criteria were major medical problems or poor physical conditions that might impede participation.

In our clinical trials, participants with stroke received bimanual robotic-primed interventions. The programs were delivered for six weeks, three days a week, and 90 min a day. Qualified occupational therapists administered the pre- and post-treatment assessments. This present research used the data for machine learning-based study of outcome prediction.

### 2.2 Outcome measures and potential predictors

Stroke recovery was measured by the ABILHAND. Participants whose improvement achieved the minimal clinically important difference (MCID) at the postintervention assessment were labeled responders, and those who did not achieve the MCID were labeled nonresponders. Both anchor-based (0.26) and distribution-based (0.35) MCIDs (26) were adopted in this study, and we constructed separate models for the two MCIDs. The MCIDs were determined in a previous study (26), where the anchor-based MCID was estimated using the SIS as the anchor, and the distribution-based MCID was estimated using the Cohen effect size benchmark. For each cutoff value, we first constructed full classification models with 14 features to predict the responder/nonresponder labels. The 14 features (i.e., the potential predictors) were of three categories: 1) demographic characteristics, including age, sex, and years of education; 2) stroke characteristics, including time since stroke, side of brain lesion, diagnosis (hemorrhagic or ischemic), and the baseline score of the National Institutes of Health Stroke Scale (NIHSS); and 3) baseline assessment scores, including scores on the Fugl-Myer Assessment of the Upper Extremity (FMA-UE), Chedoke Arm and Hand Activity Inventory (CAHAI), Wolf Motor Function Test (WMFT)-Time and -Quality, SIS-Physical function, ABILHAND, and the Medical Research Council (MRC) scale.

### 2.3 Data analysis

Two types of models were constructed: full models and parsimonious models. The full models were built with all 14 features, and parsimonious models were built with the 5 most important features. Each type of model was constructed for the two predicted targets, namely, categorical labels with an MCID of 0.35 and those with an MCID of 0.26.

For each target, four machine learning algorithms were used: logistic regression (LR), k-nearest neighbors (KNN), support vector machine (SVM), and random forest (RF). Previous studies have recommended the use of multiple algorithms to build models and compare performances (27), which we adopted in our previous study (9). The chosen algorithms differ in their computation methods and levels of complexity. LR was chosen to test the ability of a simpler algorithm in our categorization task. KNN and SVM have moderate levels of complexity and are frequently used in machine learning studies involving stroke rehabilitation (9, 12, 15, 16, 18, 27, 28). RF has the highest level of complexity and was selected to test whether algorithms with higher levels of complexity would yield better performance. In total, 32 models were constructed (four targets ×  two types × four algorithms).

**Figure 1:**
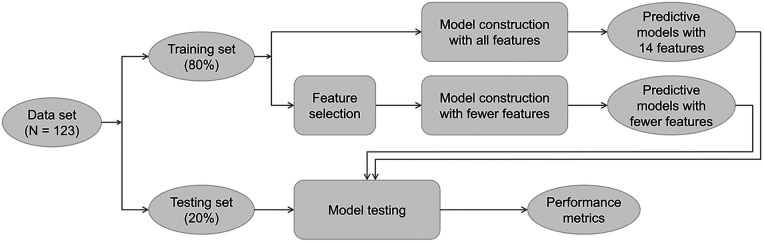
Flowchart for the machine learning data analysis.

Figure 1 presents the steps for the machine learning data analysis. The dataset included 123 data points. The entire dataset was split randomly with stratification into two sets: 80% in the training set and 20% in the testing set. The training set with all 14 features was used to build the full models. For the parsimonious models, the training set first underwent feature selection, and the five most important features were retained in model construction. Feature selection was performed by calculating the mutual information gain. After the models were constructed, the testing set was used to evaluate model performance. This approach ensured that the data used to test model performance were never used during model construction and did not influence the models.

To minimize the effect of class imbalance, we performed the synthetic minority oversampling technique to the training set (29). Except when RF was used, the data were also standardized to avoid dominating effects from numerical features on larger scales (30). Models were tuned using grid search and 10-fold cross-validation to identify optimal values for hyperparameters, i.e., values that obtained the highest classification accuracy. For LR, the procedure identified the optimal c value and maximum number of iterations. For KNN, the procedure identified the distance weight and the optimal number of neighbors. For the SVM, the procedure identified the optimal kernel and c value. For the RF, the search procedure identified the maximum depth. The values of all the other hyperparameters remained at the default values. Appendix 1 shows the hyperparameters selected for study.

Model performance was assessed with the classification accuracy and the area under the receiver operating characteristic curve (ROC-AUC). All data analyses, including descriptive statistics and the construction and validation of the machine learning models and their corresponding data preprocessing, were conducted using Python 3.10.12 software [(31), RRID: SCR_008394], with the packages numpy 1.26.4, pandas 2.2.2, scipy 1.15.3, sklearn 1.5.2 (32), imblearn 0.12.4 (33), and matplotlib 3.10.0 (34).

## 3 Results

### 3.1 Participant characteristics

The study included 123 participants with values of all 14 predictors available. The demographics, stroke characteristics, and baseline assessment scores of the participants are summarized in Table 1. Ninety participants achieved an anchor-based MCID (0.26), and 86 participants achieved a distribution-based MCID (0.35). After the dataset was randomly split, the training set included 98 participants and the testing set included 25 participants. Comparisons for individual features between the two sets were performed using independent t tests or chi-square tests. None of the comparisons yielded statistically significant differences (*p* = 0.05–0.92 for MCID = 0.35; *p* = 0.07–0.93 for MCID = 0.26).

**Table 1:** Participant characteristics.

Characteristics	Mean ± SD/Participants, No. (%)
Demographics	
Age (years)	55.43 ± 11.20
Male sex	82 (67%)
Years of education	11.64 ± 4.15
Stroke characteristics	
Left-sided brain lesion	74 (60%)
Time since stroke (months)	19.20 ± 15.66
Hemorrhagic stroke diagnosis	57 (46%)
NIHSS score	4.94 ± 2.81
Baseline assessment scores	
FMA-UE	32.40 ± 8.99
CAHAI	38.02 ± 15.68
WMFT-time	10.98 ± 5.64
WMFT-wuality	2.47 ± 0.56
SIS-physical function	55.96 ± 14.06
ABILHAND	−0.28 ± 1.03
MRC	2.74 ± 0.78

CAHAI, chedoke arm and hand activity inventory; FMA-UE, upper extremity subscale of the Fugl-Meyer assessment; MRC, medical research council; NIHSS, National Institutes of Health Stroke Scale; SD, standard deviation; SIS, stroke impact scale; WMFT, wolf motor function test.

### 3.2 Most important predictors

Table 2 shows the mutual information gains; the predictors for each target are sorted in descending order according to gain.

**Table 2:** Mutual information gains for predictors sorted in descending order.

MCID = 0.35		MCID = 0.26	
Predictor	Gain	Predictor	Gain
Time since stroke	0.04	WMFT_Time_	0.07
ABILHAND	0.04	SIS_Physical_	0.05
CAHAI	0.03	Sex	0.04
WMFT_Time_	0.02	Diagnosis	0.03
SIS_Physical_	0.01	Time since stroke	0.02
Diagnosis	0.00	Education	0.02
Education	0.00	Side of brain lesion	0.02
Age	0.00	ABILHAND	0.00
Sex	0.00	CAHAI	0.00
Side of brain lesion	0.00	Age	0.00
NIHSS	0.00	NIHSS	0.00
FMA-UE	0.00	FMA-UE	0.00
WMFT_Quality_	0.00	WMFT_Quality_	0.00
MRC	0.00	MRC	0.00

CAHAI, chedoke arm and hand activity inventory; FMA-UE, upper extremity subscale of the Fugl-Meyer assessment; MCID, minimal clinically important difference; MRC, medical research council; NIHSS, National Institutes of Health Stroke Scale; SIS, stroke impact scale; WMFT, wolf motor function test.

### 3.3 Model performance

**Figure 2:**
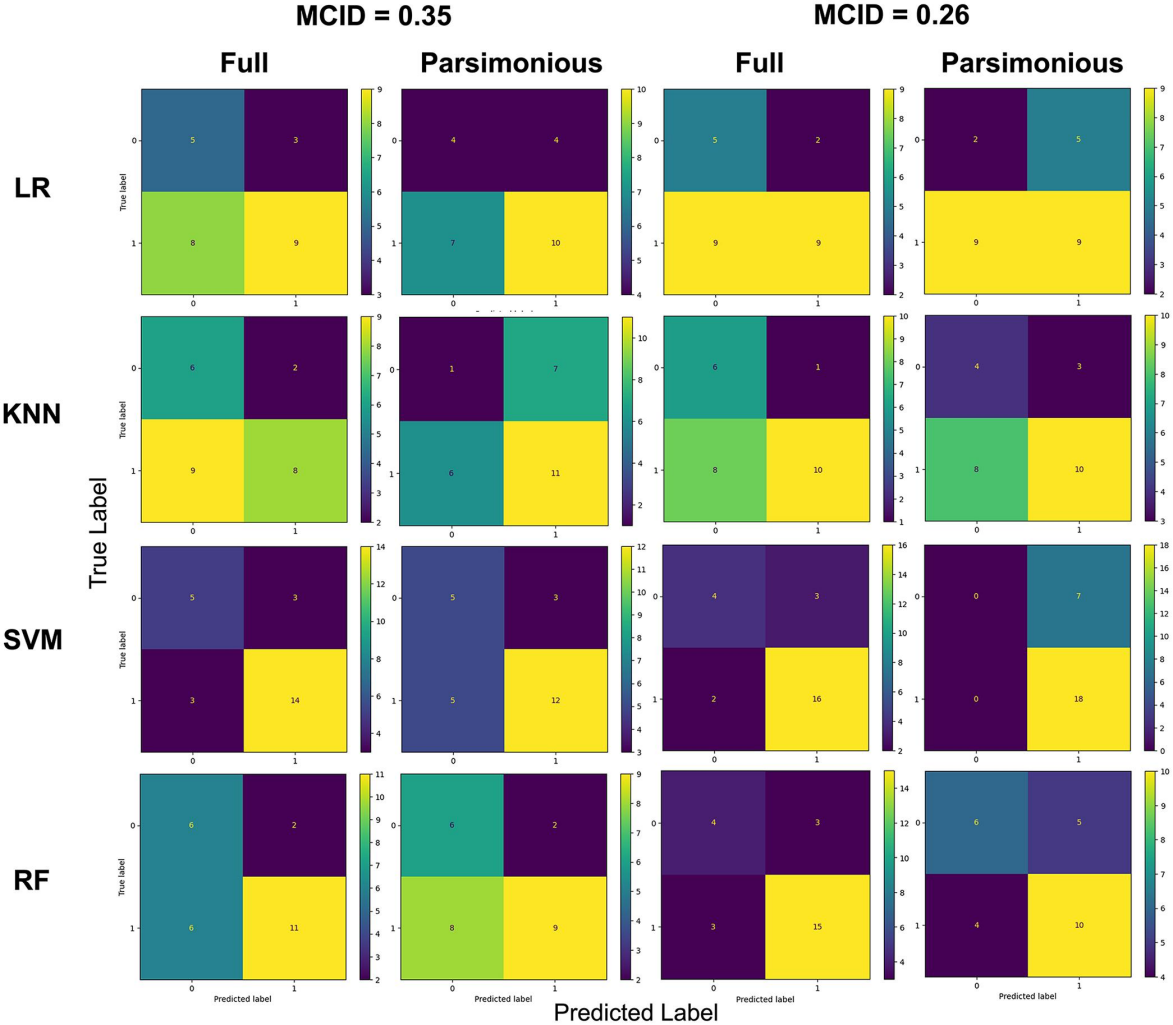
Confusion matrices for the prediction models. KNN, k–nearest neighbors; LR, logistic regression; RF, random forest; SVM, support vector machine.

Model performance is summarized in Table 3. Figure 2 shows the confusion matrices. For the models built with all 14 features, SVM performed better than the other algorithms. When recovery achievement was defined with MCID = 0.35, the best model prediction accuracy and ROC-AUC were 0.76 and 0.75, respectively. Grid search identified the radial basis function as the optimal kernel and 10 as the best c value. When recovery achievement was defined with MCID = 0.26, the best model prediction accuracy and ROC-AUC were 0.80 and 0.84, respectively. Grid search identified the radial basis function as the optimal kernel and 10 as the best c value. For the parsimonious models, SVM and RF were exhibited the best performance in terms of predicting recovery at MCID values of 0.35 and 0.26, respectively. For the SVM model, the grid search identified the radial basis function and 10 as the best hyperparameters; for the RF model, the optimal depth was 3. Compared with the full models, the parsimonious models had worse performance. This occurred across the algorithms used and across both MCID values.

**Table 3:** Model performance.

Algorithm	Metric	MCID = 0.35		MCID = 0.26	
		FULL	PARS	FULL	PARS
LR	Accuracy	0.56	0.56	0.56	0.44
	ROC-AUC	0.74	0.59	0.56	0.53
	Specificity	0.63	0.50	0.71	0.29
	Precision	0.63	0.60	0.69	0.51
	Recall	0.56	0.56	0.56	0.44
	F1-score	0.57	0.57	0.58	0.47
KNN	Accuracy	0.56	0.48	0.64	0.56
	ROC-AUC	0.51	0.52	0.75	0.60
	Specificity	0.75	0.13	0.86	0.57
	Precision	0.67	0.46	0.77	0.65
	Recall	0.56	0.48	0.64	0.56
	F1-score	0.57	0.47	0.66	0.58
SVM	Accuracy	**0** **.** **76**	**0**.**68**	**0**.**80**	0.72
	ROC-AUC	**0**.**75**	**0**.**68**	**0**.**84**	0.58
	Specificity	0.63	0.63	0.57	0.00
	Precision	0.76	0.70	0.79	0.52
	Recall	0.76	0.68	0.80	0.72
	F1-score	0.76	0.69	0.80	0.60
RF	Accuracy	0.68	0.60	0.76	**0**.**64**
	ROC-AUC	0.74	0.62	0.79	**0**.**68**
	Specificity	0.75	0.75	0.57	0.86
	Precision	0.74	0.69	0.76	0.64
	Recall	0.68	0.60	0.76	0.64
	F1-score	0.69	0.61	0.76	0.64

FULL, models built with 14 features; KNN, k-nearest neighbors; LR, logistic regression; PARS, models built with 5 features; RF, random forest; ROC-AUC, area under the receiver operating characteristic curve; SVM, support vector machine.

Bold fonts indicate the best performance values.

## 4 Discussion

In recent years, PROMs have received and increasing amount of attention as healthcare has shifted toward a patient-centered model. Extended from our previous report in which machine learning was used to predict PROMs of activity and participation in chronic stroke (9), the present study aimed to investigate predictors of patient-reported functional recovery of bilateral arm function. Our results revealed that time since stroke, WMFT-Time, and SIS-Physical function were important predictors regardless of which MCID was adopted. The baseline ABILHAND and CAHAI scores were among the most important predictors when the anchor-based MCID (0.35) was adopted to define the achievement of perceived recovery. When the distribution-based MCID (0.26) was adopted, sex and stroke diagnosis were predictive.

In both the subacute and chronic stages, the duration between stroke occurrence and the initiation of rehabilitation has been identified as an important predictor of rehabilitation outcome. Shorter delays are associated with better functional recovery among individuals in the subacute stage of stroke (11, 35, 36). Similarly, in people with chronic stroke, the duration between stroke occurrence and rehabilitation initiation was reported to be an important predictor of postintervention upper-limb motor function (27, 37) and patient-reported postintervention improvement in activities of daily living (9). Our study used machine learning, which does not yield a directional result. *post hoc* analysis revealed that the participants categorized as responders had shorter intervals between stroke onset and rehabilitation initiation.

A recent study investigated predictors of posttreatment improvement in upper-limb motor function as measured by the FMA-UE in people with chronic stroke (38). The time since stroke was found to be an important predictor, but the effect was in the opposite direction compared to our findings. In their logistic regression analysis, people with stroke who had a longer time after stroke were more likely to achieve the MCID after robotic therapy. The differences in findings most likely reflect the different characteristics of the stroke populations in the two studies. Our study excluded people with FMA-UE scores of 10 and lower, and the participants demonstrated mild to severe baseline motor function, with an average FMA-UE scores of 32 points. The participants in the study of Dimyan et al. (38) had more severe motor impairment, with an average FMA-UE of 19.8. These findings are consistent with those of Lee et al. (39), who reported that a subgroup of more severely impaired participants experiences greater benefits from robotic therapy. Furthermore, in our study, the participants were in the subacute to chronic stages, with an average duration since stroke onset of 32 months. In the Dimyan et al. study (38), the participants had chronic stroke, with the average time since stroke onset being 5.4 years (64.84 months). The association between time since stroke onset and stroke recovery after therapy may not be linear. This discrepancy highlights the importance of including time since stroke in future research to further understand this factor in stroke recovery.

WMFT-Time, but not WMFT-Quality, was an important predictor of patient-perceived recovery. This finding may reflect the aspects of motor performance that people with stroke perceive as important in stroke recovery. Our *post hoc* analysis revealed that the mean WMFT-Time scores were lower in the responder group than in the nonresponder group, although the differences were not statistically significant. We speculate that the ability of people with stroke to complete tasks more quickly contributes to their confidence in upper limb recovery and fosters a stronger sense of ability to perform bilateral arm activities. The results may provide clinicians with better tools for identifying responders to improvements in perceived bilateral functional arm use (i.e., the ABILHAND) after bimanual robotic therapy for 6 weeks among people with stroke in the subacute or chronic stages. Interestingly, these findings are consistent with those of a recent study investigating predictors of recovery in upper-limb motor function using the clinician-rated FMA-UE (38), suggesting that the duration required to complete a test item may influence therapist rating of the performance of upper limbs.

Unsurprisingly, the SIS-Physical score was among the most important predictors of recovery. The SIS-Physical is also a PROM related to stroke recovery and may tap into similar values and perspectives of people with stroke as the ABILHAND. Previous research has revealed a strong correlation between ABILHAND scores and SIS-Physical scores (26). Our findings support this relationship. Notably, although the scales are highly correlated, they have different assessment scopes. SIS-Physical is a more comprehensive scale and evaluates a broader range of physical functions; it measures strength, function, and mobility and is particularly useful in understanding the physical limitations and challenges faced by people with stroke. The ABILHAND specifically measures bilateral arm function, including dexterity and manual ability, and provides insights into the ability of people with stroke to manage bilateral arm tasks in daily life (5). The ABILHAND is more closely related to the treatment targets of our robotic therapy rehabilitation program.

The baseline ABILHAND score was a strong predictor of patient-perceived recovery when the anchor-based MCID was adopted. In our previous study, the baseline scores of a given PROM of activities of daily living also stood out as important predictors for achieving MCID in the PROM itself (9). This finding was in line with the literature, as postintervention upper-limb motor function can be predicted by the baseline scores of a given measure itself when analyzed via machine learning (27, 37) and traditional statistical methods (39). The CAHAI was also a strong predictor of perceived recovery as measured by the ABILHAND. Although the CAHAI is a clinician-rated scale and may not accurately capture the perspectives of people with stroke, this performance-based clinician-rated scale focuses on bilateral arm function and movement, similar to the ABILHAND. These results further demonstrate the multidimensionality of a patient-reported outcome.

Defining patient-perceived recovery of hand function via the two MCIDs yielded similar, but not identical results with respect to the most important predictors. Using the anchor-based MCID, the most important predictors included the time since stroke, SIS-Physical score, WMFT-Time, baseline ABILHAND score, and baseline CAHAI score. Using the distribution-based MCID, the most important predictors included the time since stroke, SIS-Physical score, WMFT-Time, sex, and stroke diagnosis. The differences in the important predictors between the two approaches most likely reflect the estimation approaches and consequent clinical implications of the two MCIDs. The anchor-based MCID is estimated via an external measure of perceived change and is most closely associated with patient-perceived outcomes and clinician-rated outcomes (40, 41). It is therefore not surprising that the baseline scores of the ABILHAND and the CAHAI were found to be important predictors of hand recovery, as defined by an anchor-based MCID. In contrast, the distribution-based MCID is estimated via the score distribution from a sample and therefore is linked to sample characteristics (42, 43). In our analysis, the sample characteristics that stood out as the most important predictors of achieving hand recovery were sex and stroke diagnosis.

To our knowledge, this is the first study to investigate the predictors of bilateral arm function recovery measured by a PROM after robotic stroke rehabilitation. Previous studies using robotic therapy investigated predictors of recovery measured by clinician-rated upper-limb motor function (38, 44, 45). Hsu et al. (45) reported that baseline motor impairment (measured by the FMA-UE) predicted motor function after robotic therapy. Although we also included the FMA-UE score as a potential predictor, it was not found to be an important predictor in our analysis. This finding indicated that results of outcome prediction study may differ with regards to clinician-rated outcomes and patient-reported outcomes in stroke rehabilitation. Furthermore, in line with our findings, age was not found to be a predictor of upper-limb motor function recovery after robotic therapy in the study by Cecchi et al. (44). These findings indicate that robotic therapy has the potential to yield treatment benefits irrespective of the age of people with stroke.

The models constructed herein exhibited fair to good performance. In line with the literature (12, 15–18, 28) and our previous study (9), SVM was found to exhibit the best performance. It is recommended that algorithms with midlevel complexity be included in future models for predicting stroke-rehabilitation outcomes. Notably, in one of our analyses (when recovery was defined by a cutoff of 0.26), although SVM yielded good classification accuracy (0.72), the ROC-AUC was fair (0.58). RF yielded slightly better results, with a lower accuracy but more balanced accuracy and ROC-AUC. Our choice of algorithms was based on the findings of our prior study (9) and other similar reports. In the study by Chen et al. (9), the more complex RF algorithm did not yield classification results superior to or comparable to those of other algorithms. This finding was potentially attributed to the larger number of potential predictors included in that study and the small sample size. Tree-based models are prone to overfitting when they grow too deep, which could still occur in RFs despite their lower risk. Overfitting in tree-based models, including RF, is especially likely to occur when the dataset includes many features and a smaller number of observations (46). Compared with Chen et al. (9), the current study included only 14 potential predictors with a similar sample size. This may have resulted in the improved performance of the RF in this dataset. These findings support the recommendation to use multiple algorithms in rehabilitation data analysis (9, 17).

For three out of the four targets, our parsimonious models exhibited worse performance than the full models. A decrease of 0.08–0.16 was observed in the performance metrics. This decrease may reflect the complexity and variability in patient-perceived stroke recovery. Previous studies have identified a variety of important predictors for PROMs in stroke rehabilitation, including baseline scores of activities of daily living reported by people with stroke (9), baseline motor function (9, 24), demographic characteristics (3), and stroke characteristics (3). PROMs may be complex constructs, and the factors associated with them may be multidimensional. Another possibility is that the 14 features did not allow feature selection to refine to smaller, more powerful sets of the most important features. As shown in Table 2, the individual features had small mutual information gains. There may be other potentially highly important predictors that we did not examine in this study.

Notably, despite their reduced performance, the parsimonious models can still provide valuable insights into identifying potential responders, especially when some values of the 14 predictors are unavailable or missing for a new participant. In such cases, clinicians could use the most important predictors to estimate the possible prognosis. Clinically, it may be difficult to complete a comprehensive assessment in one session. In our clinical setting, completing two to three assessment tests in the first encounter is usually doable. Selection of five most important features for study is feasible. In our previous study (9), we tested models built with four to six most important features, and the results yielded similar model performances. Clinicians from other clinics could also build customized models depending on data available in their clinics. Although the process requires the clinician to interact with Python code or specialized programs on a computer, it can be accomplished with minimal clinician training and relatively modest technology requirements.

### 4.1 Study limitations

This study has several limitations. First, as with many other rehabilitation studies, the sample size was limited for machine learning-based study. Our study results should be interpreted with caution. However, we adopted additional measures to minimize the potential bias due to the size of study. We balanced the sample via synthetic minority oversampling technique and standardized the data to minimize the effects of differences in scales. We also reserved an unused subset of data for model evaluation to ensure that the testing set did not affect model construction. The single split may have limited model learning; however, we applied 10-fold cross-validation on the training set during construction to minimize the impact while still leaving a hold-out set for testing. Additionally, with a limited sample size and a relatively large number of features, machine learning models may overfit, thus decreasing their generalizability. When the testing set is included in model training, the testing scores in the performance evaluation are optimistic (47). Having a novel testing set helps identify overfitting when it occurs. Another potential consequence of the small sample size is the limited amount of the testing data. With the 80%–20% split, the testing set had 25 data points. We have attempted a 75%–25% split during data analysis, but this did not yield better model performance.

Further, because this study is a secondary data analysis, we may have missed some potentially important predictors that could help improve model performance. PROMs are complex constructs and may require a wider range of variables for a more comprehensive prediction. Although hand function is a focused target, the ABILHAND, which is a PROM, can still require a wider range of perspectives of people with stroke for prediction. There could also have been some redundant features, reflected by the very low mutual information gains. The close-to-zero gains indicate that these features each contributed very little to our prediction of rehabilitation outcome in ABILHAND. Interestingly, the parsimonious models, which did not include any feature with close-to-zero gains, did not perform better. This finding indicated that some important features may have been missing from our dataset. Further research is needed to study features that reflect stroke rehabilitation and relevant cultural and personal beliefs such as measures of self-efficacy and rehabilitation psychology. Notably, when more features are included, machine learning analysis is prone to dimensionality. This limitation could be overcome by dimension reduction and by increasing the sample size. However, increasing the sample size may be difficult in healthcare and rehabilitation studies because of the limited availability of people with stroke and because of the strict inclusion criteria. Dimension reduction may be required if more features are included. It is therefore recommended that future studies with more features perform feature selection or adopt methods such as linear discriminant analysis and construct smaller models to accompany the full models.

Of note, several factors may have limited generalization of the study results beyond the scope of the study. First, the study participants in the present study received bilateral robotics-primed interventions. The study findings may not be generalized to individuals receiving other rehabilitative programs. Further, PROMs may be influenced by cultural beliefs. Results of the present study warrant verification in other cultures. Third, selection of machine learning methods and parameters during model construction require careful considerations and the study results may be specific to the study sample and the method of machine learning.

## 5 Conclusion

In this study, we used machine learning approaches to predict postintervention recovery of bilateral arm function after robotic stroke rehabilitation. We identified sets of important predictors for the anchor-based MCID and the distribution-based MCID. Early initiation of the therapy, being able to complete a task quickly, and being better able to self-perceive physical function are critical aspects of regaining self-rated function of the bilateral upper limbs in daily activities after six weeks of robotic stroke rehabilitation. The findings may inform clinicians about characteristics of individuals with stroke who are more likely to benefit from robotic therapy for enhanced perception of bilateral arm function in daily activities. Further research is needed to validate the findings based on larger samples.

## Data Availability

The raw data supporting the conclusions of this article will be made available by the authors, without undue reservation.

## Appendix

Hyperparameters

**Table:**

Algorithm	Hyperparameter	MCID = 0.35		MCID = 0.26	
		Full	Pars	Full	Pars
LR	c	1	100	1	0.001
	max_iter	100	100	100	100
KNN	n_neighbors	6	1	12	11
	weight	distance	uniform	distance	uniform
SVM	c	10	10	10	0.001
	kernel	rbf	rbf	rbf	poly
RF	max_depth	4	8	6	3
